# Supplementary figures and images for: Structural basis of EHEP-mediated offense against phlorotannin-induced defense from brown algae to protect akuBGL activity
Source: eLife. 2023 Nov 1;12:RP88939. doi: 10.7554/eLife.88939 (PMC10619976; doi:10.7554/eLife.88939)

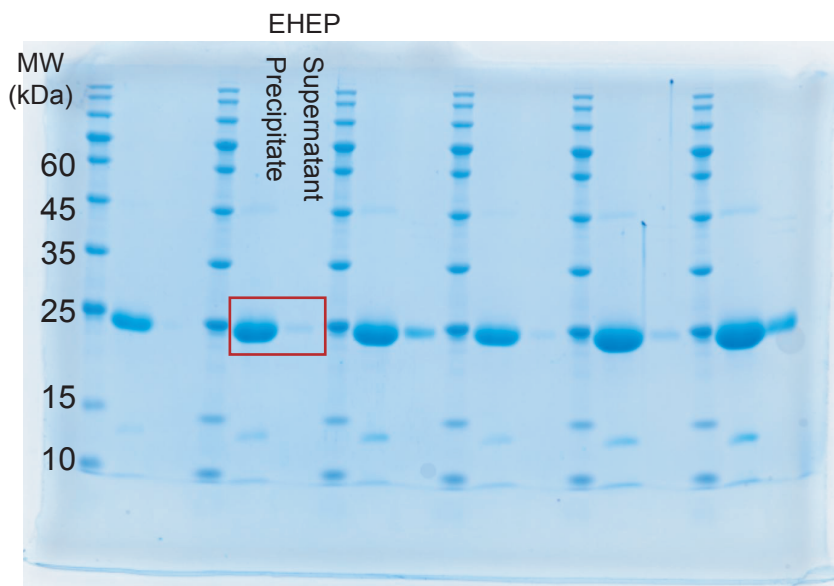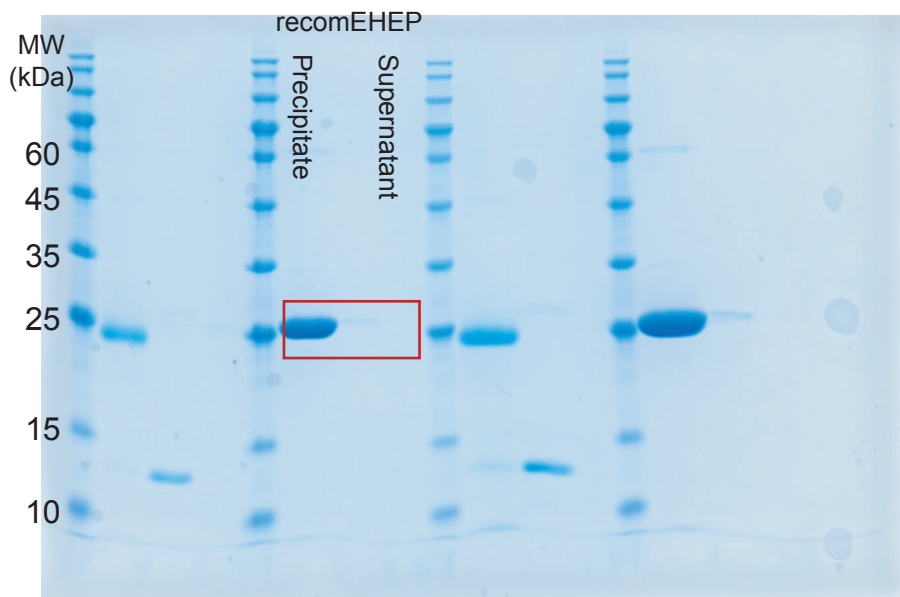

Supplement: Figure 2—figure supplement 1—source data 1. [file elife-88939-fig2-figsupp1-data1.zip › Figure 2—figure supplement 1B-uncropped gel.pdf]

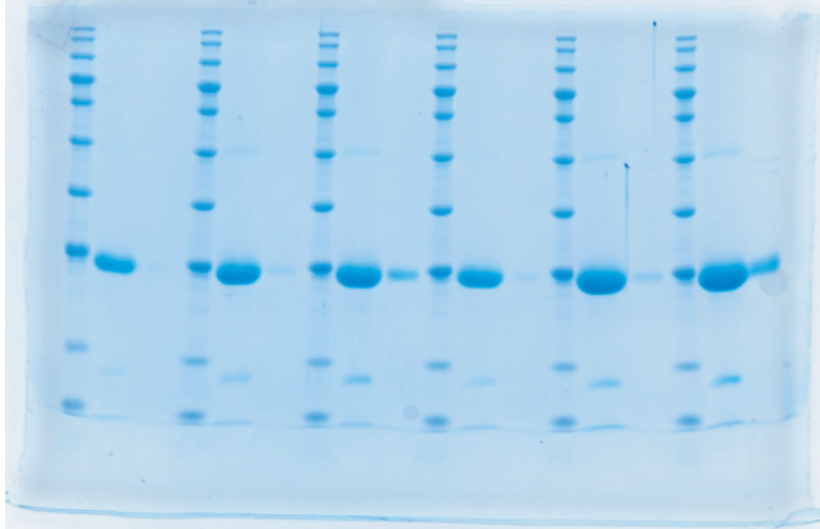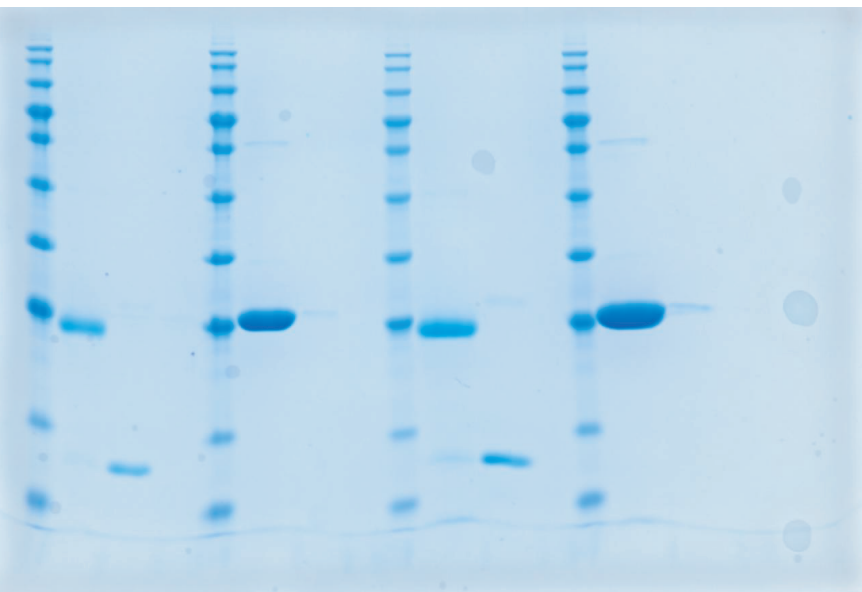

Supplement: Figure 2—figure supplement 1—source data 1. [file elife-88939-fig2-figsupp1-data1.zip › Figure 2—figure supplement 1B-raw gel.pdf]

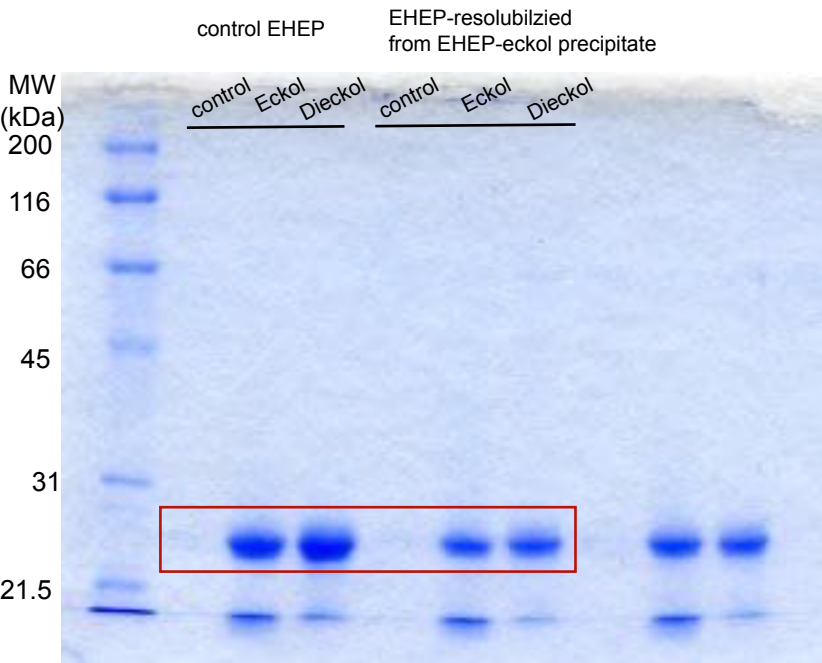

Supplement: Figure 3—figure supplement 1—source data 1. [file elife-88939-fig3-figsupp1-data1.zip › Figure 3—figure supplement 1D-uncropped gel.pdf]

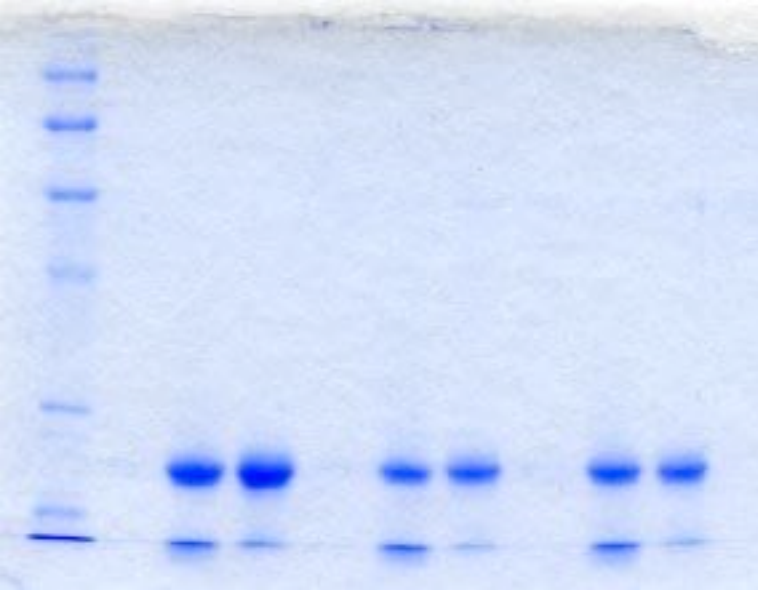

Supplement: Figure 3—figure supplement 1—source data 1. [file elife-88939-fig3-figsupp1-data1.zip › Figure 3—figure supplement 1D-raw gel.pdf]

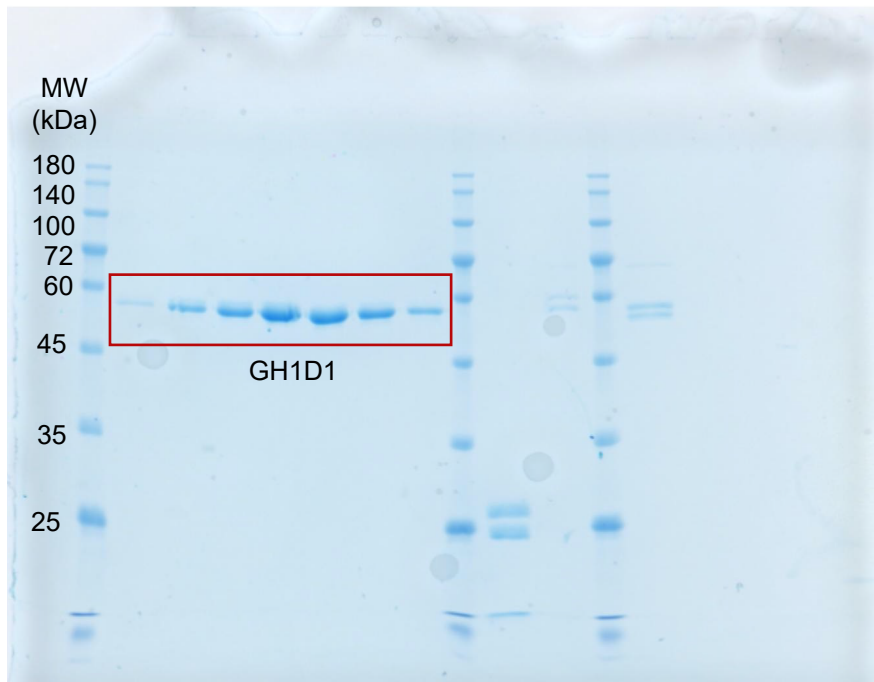

Supplement: Figure 4—figure supplement 1—source data 1. [file elife-88939-fig4-figsupp1-data1.zip › Figure 4—figure supplement 1-uncropped gel.pdf]

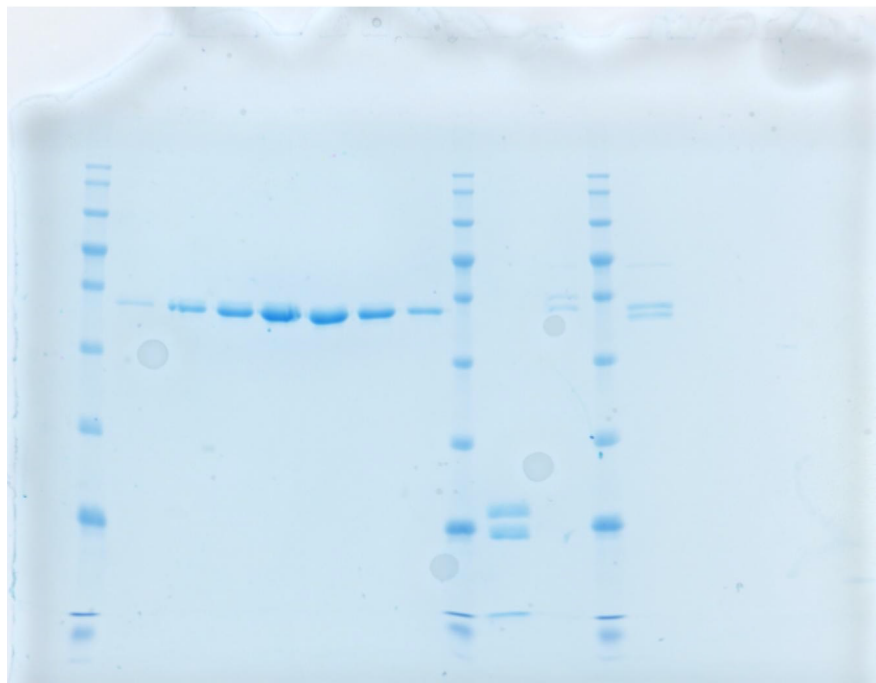

Supplement: Figure 4—figure supplement 1—source data 1. [file elife-88939-fig4-figsupp1-data1.zip › Figure 4—figure supplement 1-raw gel.pdf]
