# Supplementary material for: Structural basis of EHEP-mediated offense against phlorotannin-induced defense from brown algae to protect akuBGL activity
Source: eLife. 2023 Nov 1;12:RP88939. doi: 10.7554/eLife.88939 (PMC10619976; doi:10.7554/eLife.88939)
Supplement: Supplementary file 1. [file elife-88939-supp1.docx]

**Supplementary Table 1. Primers used in this study**

| plasmid | primers (5’-3’) |
| --- | --- |
| EHEP-20-28M | Forward: GGGAATTCCATATGGCCGTTAACCTGTGCACCC  Reversed: CCGCTCGAGTTATTTGCCGACCAGGACGTTTCC |
| GH1D2-pET-32a | Forward: CTGTATTTTCAGGGCGATAAATTTCCGGCCAATTTTACCTTT  Reversed: GAGCTCGAATTCGGATCCTTAATCGGTCAGACCATTATTGGCAATC |
